# Exposure to firearm injury and suicide in a rural Pennsylvania county: implications for mental and behavioral health

**DOI:** 10.1007/s10865-024-00529-8

**Published:** 2024-10-28

**Authors:** Richard Stansfield, Daniel C. Semenza, Devon Ziminski

**Affiliations:** 1 Department of Sociology, Anthropology and Criminal Justice, Rutgers University – Camden, 405-407 Cooper Street, Camden, NJ 08102 USA; 2 New Jersey Gun Violence Research Center, Rutgers University, New Brunswick, USA

**Keywords:** Firearm injury, Violence, Suicide, Mental health, Behavioral health, Rural

## Abstract

To evaluate the association between self-reported gun violence exposures and mental health and behavioral health indicators in a rural population. Using cross-sectional survey responses from 630 residents of a rural county in Pennsylvania, logistic regression models estimate the likelihood of residents indicating moderate or severe levels of health outcomes as a function of gun violence exposure. We control for a series of variables related to gun ownership, behavior, history with firearms and demographic characteristics. Personal firearm victimization was associated with self-reported depressive symptoms and interrupted sleep. Secondary exposure to firearm violence, particularly exposure to friends attempting or completing a suicide, was associated with higher odds of reporting severe levels (14 days or more in the past month) of depressive symptoms, anxiety, and poor sleep. For firearm suicide involving a friend or family member, the odds of reporting severe levels of all three outcomes are 3 times greater (OR 2.984, 95% CI 1.457–6.108). For each additional firearm exposure, the odds of experiencing severe levels of mental health and sleep difficulties are 1.4 times greater (OR 1.384, 95% CI 1.115–1.720). Cumulative exposures also increase the odds of reporting binge drinking and drug use. Firearm violence exposure was associated with adverse health indicators in this rural population. Approaches to counter the effects of cumulative firearm exposure and firearm suicide exposure, including reinvigorating community spaces and strengthening social supports, may help to reduce mental health burden in rural communities.

## Background

A growing body of evidence makes clear that exposure to firearm violence is associated with poorer mental, physical, and behavioral health among individuals (Semenza et al., 2023; Semenza et al., 2024; Smith et al., 2020; Song et al., 2022, 2023; Turner et al., 2019; Vasan et al., 2021) and throughout entire communities (Semenza & Stansfield 2021a, b; Semenza et al., 2023b). In two recent longitudinal studies using case–control matches of commercial health insurance claims, researchers found that children and adolescent survivors of firearm violence experienced substantial increases in pain, psychiatric, and substance use disorders in the year following firearm injury relative to controls (Song et al., 2023). Similarly, healthcare spending, psychiatric disorders, and substance use disorders all increased among adult survivors of firearm injury (Song et al., 2022). Accounting for the broad health-related consequences of firearm violence particularly due to quality-of-life losses among those injured by firearms and their families, the total economic toll of firearm injuries in the United States (US) is estimated to be about $557 billion each year or roughly 2.6% of gross domestic product (Song, 2022).

The health burden of firearm exposure is not limited to direct injury. People can also be exposed to gun violence by knowing a family member or friend that has been shot or killed (i.e., secondary survivors) or hearing about or witnessing shootings among those outside of immediate social circles in local areas (i.e., community violence) (Magee et al., 2023). For instance, in a nationally representative, cross-sectional study of Black adults in the US, Semenza et al. (2024a) found that being threatened with a firearm and knowing a family member or friend who had been shot was associated with reporting both lifetime suicidal ideation and suicide attempt. Family members of firearm injury survivors and shooting decedents experience drastic increases in psychiatric disorders, healthcare spending, and mental health visits after a loved one is injured (Song et al., 2022). People also often experience multiple types of gun violence exposure (i.e., cumulative exposure) that include direct, secondary, and community-level experiences. For example, roughly 12% of Black adults and 13% of American Indian/Alaksa Native (AI/AN) adults living in the US have experienced three or more types of gun violence exposure in their lifetime (Semenza et al., 2024b). Cumulative exposure is particularly associated with degraded health in a frequently “dose–response” manner such that a greater number of exposure types is linked to more sleep problems, worse self-rated health, and more days of poor mental and physical health each month (Semenza et al., 2023, 2024).

Despite expanding research on the broad health-related consequences of firearm injury across diverse forms of exposure, there remain two notable limitations to this work. First, few studies have focused specifically on the relationship between different types of firearm exposure and health among rural populations (Mitchell et al., 2021; Slovak & Singer, 2001). The majority of individual-level and ecological studies on gun violence exposure and health have examined large urban areas (Buggs et al., 2022; Magee et al., 2022; Semenza & Stansfield, 2021a; Semenza et al., 2023b; Smith et al., 2020). Second, researchers have focused predominantly on health ramifications of exposure to interpersonal firearm violence, rather than self-directed firearm violence (i.e., attempted or completed suicide). Together, these are two critical limitations since rural communities experience higher overall rates of firearm death than their urban counterparts and those rates are driven by much higher rates of firearm suicide (Reeping et al., 2023). To address these limitations, we analyzed the associations between numerous interpersonal and self-directed firearm violence exposures (individual and cumulative) and diverse health outcomes among residents of a rural county in Pennsylvania.

## Data and methods

The study setting is a rural county of the Commonwealth of Pennsylvania, as defined by the Center for Rural Pennsylvania (rural.pa.gov). The county spans 617 square miles with a density of 267 people per square mile, though this varies significantly across the county. Recreational firearm use is common in the County, with hunting and recreational shooting being popular among residents and tourists. The county was awarded a grant under the Gun Violence Investigation and Prosecution (GVIP) Grant Program, which provided the foundation for the development of a Community Partnership for Gun Safety. Among many initiatives, the group was tasked with undertaking a community needs assessment, including gathering information about gun violence and injury via official data sources and a community survey.

The study’s survey assessed a wide range of firearm-related behaviors, questions about firearm ownership and history growing up with firearms, exposure to firearm injury, and support for community interventions (questions used for current study displayed in Appendix 1). The survey was administered via the Survey Monkey online survey platform between September and November 2023. Residents were encouraged to complete the survey via QR codes and online links to the survey were promoted throughout local newspapers, social media, community organizations, and government listservs. Although a total of 719 respondents completed the survey, 89 responses were removed from the sample because either the respondents did not reside in the county, or responses were missing across a majority of questions. Care was taken to ensure respondents were residents of the county using screening questions such as the nearest intersection, cross-checked with self-reported zip-codes. In addition, IP addresses were analyzed to ensure obvious fraudulent responses and duplicate entries were removed. This left a final sample of 630 respondents for analysis. Respondents were not reimbursed for their participation in the survey. Prior to the analysis of non-identifiable anonymous responses, ethical approval was sought and exempted.

### Dependent variables

To capture multiple dimensions of behavioral and mental health, the survey included validated questions related to quality of life developed and used in the Behavioral Risk Factor Surveillance System (Kobau et al., 2004). Respondents were asked the following questions, “During the past 30 days, for about how many days have you felt sad, blue, or depressed?”, “During the past 30 days, for about how many days have you felt anxious, tense, or worried?”, and “During the past 30 days, how many days have you had trouble falling asleep or staying asleep or sleeping too much?” Responses options included the following categories: 0 days, 1–3 days, 3–7 days, 7–14 days, and 14 or more days. The Centers for Disease Control and Prevention (CDC) often categorize these symptoms of generalized anxiety as mild, moderate, or severe (Villarroal & Terlizzi, 2020), with symptoms that last for 7 days or more considered moderate, and 2 weeks or more indicating a more severe problem that can interfere with everyday functioning. For our analyses, responses were dichotomized so that we could differentiate between both moderate levels and severe levels of symptoms.

Two additional behavioral health outcomes were included in the survey to capture people’s level of alcohol and drug use. These questions were again informed by existing measures used in the BRFSS but modified in conjunction with addiction health professionals in the county (Bohm et al., 2021). To capture problematic binge drinking, respondents were asked, “How many times during the past 30 days did you have [5 or more for men, 4 or more for women] drinks on an occasion?”. Responses were dichotomized so that weekly or more frequently than weekly binge drinking was equal to 1, and less than weekly, monthly or never was equal to 0. To capture drug use, respondents were asked, “During the past 30 days, has anyone told you that they were concerned about your drug use?”, with responses limited to “yes” or “no”.

### Firearm injury exposure

Several key independent variables were created from the survey responses, broadly capturing residents’ exposure to firearm injury in terms of direct personal exposure, having friends or family exposed to firearm injury, and exposure in the community (Semenza et al., 2024a, b; Semenza et al., 2023). We include dichotomous variables indicating whether someone has ever been the victim of a violent crime with a firearm, whether someone has been the victim of a violent crime without a firearm, whether a person knows a friend or family member that has been the victim of a crime with a firearm, knows a friend or family member that has attempted or completed a suicide with a firearm, hearing about a shooting in their community, and hearing about a firearm suicide in their community.

In addition to exploring the independent associations of these firearm exposures to health outcomes, recent research suggests that cumulative gun violence exposure may be significantly associated with mental and behavioral health outcomes (Semenza et al., 2024). As such, we also created a cumulative firearm violence exposure measure, representing the count of incident types a person has experienced ranging from 0 to 6 (personal attack with a firearm (ever), friend or family victim of an attack with a firearm, friend or family use of a firearm with attempted or completed suicide, hearing about a shooting in the community, hearing about a person attempting or completing a suicide with a firearm in the community).

### Control variables

Controls include a series of dichotomous variables indicating whether a respondent currently owns a firearm, currently has a concealed carry license, grew up in a home with a firearm, is part of gun club or organization, whether a person has ever received any firearm safety training, and whether a person has ever received suicide prevention awareness training. Residents were also asked to rate their agreement with the following statement, “Your community is generally a safe place to live,” measured on a 5 point Likert scale where 5 indicates strongest agreement.

To account for one’s personal economic situation, respondents were asked to rate their agreement on the following statement, “Do you feel like you have enough money to meet your financial needs?” where 5 indicates strong agreement. Our analyses include measures for people who have lived in the county for less than 5 years (reference), 5–10 years, and more than 10 years. Finally, all analyses controlled for a range of demographic factors including self-selected gender, race, ethnicity, college education, any prior or current service in the military, and age measured in 10-year categories.

### Analytical strategy

To test the association between gun violence exposure and health outcomes, a logistic regression was first used to model the likelihood of moderate and severe self-reported anxiety, depressive symptoms, and sleep difficulty. Each measure of gun violence exposure was entered into a model one at a time to test the independent association of gun violence exposures on each of the health outcomes. A measure of cumulative gun violence exposure, as described above, was also used to estimate the change in odds of reporting mental and behavioral health difficulties for each additional exposure reported.

Given that depressive symptoms, anxiety, and sleep difficulty can often co-occur, our next analysis combines all three of these health outcomes. A count of moderate and severe health outcomes were created, ranging from 0 to 3, where for moderate combined health outcomes, 0 would indicate a respondent reported less than 7 days feeling sad, feeling anxious and difficulty with sleep. A score of 3 would indicate more than 7 days in the prior month feeling sad, feeling anxious, and having sleep difficulty. We created a combined measure of severe mental health and sleep outcomes too, where a score of 3 would indicate a respondent reported more than 14 days experiencing symptoms for each outcome. To assess the likelihood of a respondent reporting all three outcomes, relative to other categories, an ordered logistic regression model was estimated.

Finally, logistic regression models were also used to model the association between gun violence exposure and regular binge drinking and concerns about drug use. For ease of interpretation, all Tables report Odds Ratios (OR) and Confidence Intervals (CI) for our measures of gun violence exposure only. As is common in survey research, some missing data on individual items dropped the analytic sample by 18%, down from 630 respondents to 513. T-tests revealed that missing respondents were not missing systematically and did not differ significantly along measures of gun violence exposure or history and ownership of firearms.

Prior to analysis, the demographic characteristics of survey respondents were compared to county-wide estimates. These county parameters were derived from the American Communities Survey 2022. Survey respondents were generally more likely to be male, White, college-educated, and older than the county average. As an example, 49% of the county were female in 2022, compared to just 38.5% of the survey’s respondents. The county is also 76% non-Hispanic white, lower than the 84% of respondents who self-identified as non-Hispanic white. Just under half of respondents reported a college degree, but county-wide estimates suggest just 35% of residents have a 4-year degree or higher. Post-stratification weights were applied to match county parameters for gender, race, ethnicity, age, military veteran status, and college education. Weighted survey estimates were then used in analysis.

## Results

Table 1 displays the weighted and unweighted sample characteristics, revealing a population with high estimates of gun ownership and experience growing up in a home with firearms. As an example, 53% of respondents report personally owning at least one firearm in the sample, and almost 43% of respondents indicate being a member of a gun club or organization, reflective of the large presence of hunting activity and membership in the county. Data also reveals a high degree of exposure to firearm injury or violence broadly, driven in part by more than half of respondents aware of shootings or suicides in their community at large. Similar to national estimates of adults (Ballard, 2018), almost a third of respondents know someone who has died by suicide, and almost 17% of respondents also report knowing a friend or family member who has attempted or completed a suicide with a firearm. More than 40% know someone who has ever been the victim of a firearm-involved violent crime.

**Table 1:** Sample characteristics (N = 630)

Variable	Weighted %	Unweighted %	n
Sad, blue or depressed (14 days or more)	3.0%	3.1%	20
Worried, tense or anxious (14 days or more)	5.0%	4.9%	31
Not enough rest or sleep (14 days or more)	7.5%	7.7%	49
Drug use concerns	12.0%	12.4%	78
Frequent binge drinking	8.4%	9.8%	62
Ever a victim of violent crime with a firearm	25.1%	26.5%	167
Ever a victim of violent crime without a firearm	43.4%	40.8%	257
Ever a victim of non-violent crime	50.5%	49.5%	312
Friend or family victim of firearm attack	44.9%	43.3%	273
Friend or family firearm suicide	16.8%	17.8%	112
Friend of family suicide completed (any means)	27.9%	29.7%	187
Community: heard of violence victims	60.1%	61.6%	388
Community: heard of suicide	52.3%	51.4%	324
Personal gun ownership	53.0%	56.0%	353
CCW license holder	55.6%	58.6%	369
Grew up with firearms in home	64.0%	67.2%	423
Firearm club member	42.9%	44.8%	282
Received firearm safety training (no suicide)	35.2%	36.4%	229
Received firearm safety training (w/ suicide)	22.0%	26.2%	165
Residence: 0–5 years	23.3%	23.4%	147
Residence: 5–10 years	13.8%	14.1%	89
Residence: over 10 years	62.9%	62.4%	393
Age: under 35 years old	25.2%	25.4%	160
Age: 35–64	54.8%	54.4%	343
Age: 65 +	20.0%	20.2%	127
Female	49.0%	38.5%	243
Non-hispanic white	76.0%	84.0%	529
College	35.0%	48.9%	308
Military	6.0%	20.0%	126
Agree have enough money	53.8%	55.7%	351

*Cumulative firearm exposure represents a count of the following life time exposures: ever the victim of violent crime with a firearm; knowing a friend or family member that was the victim of a violent crime with a firearm; knowing a friend or family member that attempted a suicide with a firearm; knowing a friend or family member that completed a firearm suicide; hearing about a shooting in the community; hearing about someone intentionally shooting themselves in the community. (range = 0–6 exposures)

Table 2 displays the results of logistic regression models estimating the likelihood of reporting moderately high levels of behavioral and mental health outcomes, defined as 7 days or more reporting anxiety, depressive symptoms, and poor sleep. The displayed results focus on these associations between exposure and health. In multivariate analyses accounting for all demographic confounders, we found that the odds of victims of a violent crime involving a firearm reporting poor sleep were higher by a factor of 1.8. Victimization with a firearm was also particularly consequential for the odds of reporting feelings of sadness and depressive symptoms (Table 2, Model 2). In separate analyses, not reported in table, all three types of personal victimization were separated into incidents that happened in the past year versus incidents that happened longer than a year prior but given that such a small number of people reported *recent* victimization, confidence intervals for these estimates are quite wide.

**Table 2:** Logistic regression modeling mental health and sleep outcomes as a function of firearm violence exposure (*n* = 513)

Exposures	Model 1	Model 2	Model 3
	Anxious, worried, tense	Sad, blue, depressed	Poor sleep
*Personal victim*^+^			
Ever firearm violence	1.664 (.650) [0.774–3.576]	**3.727 (1.637) [1.576–8.815]**	**1.801 (.505) [1.040–3.122]**
Any violence w/out firearm	1.288 (.338) [0.771–2.153]	2.028 (.809) [0.928–4.432]	1.458 (.348) [0.913–2.328]
Non-violent crime	1.480 (.346) [0.935–2.343]	1.329(.502) [0.629–2.805]	**1.650 (.391) [1.037–2.624]**
*Friends or family*			
Violent victim firearm	1.073 (.361) [0.555–2.073]	1.197 (.451) [0.572–2.506]	0.895 (.211) [0.563–1.420]
Firearm suicide	**2.596 (.998) [1.222–5.516]**	**3.089 (1.329) [1.329–7.181]**	**2.441 (.713) [1.376–4.328]**
Died by suicide (Any means)	1.723 (.573) [0.897–3.307]	1.218 (.501) [0.543–2.731]	1.669 (.620) [0.806–3.455]
*Community*			
Heard about shooting	0.719 (.264) [0.350–1.475]	0.639 (.254) [0.300–1.394]	**2.135 (.571) [1.264–3.605]**
Heard about suicide	1.412 (.491) [0.714–2.793]	1.634 (.666) [0.735–3.632]	1.280 (.307) [0.799–2.049]
*Cumulative*			
Total firearm exposures	**1.189 (.098) [1.011–1.399]**	**1.225 (.116) [1.018–1.474]**	**1.285 (.165) [1.000–1.652]**

All models control for respondent age, gender, race, ethnicity, veteran status, education, financial need, length of residence in the county, firearm ownership, concealed carry license, growing up with firearms in the home, membership in gun clubs, and firearm safety training

^+^Each of the personal victimizations were broken down into recent victimizations happening within the previous year, and earlier experiences. Recent victimization (relative to no recent experience, and relative to no experience ever) was associated with higher odds of reporting moderate and severe levels of depressive symptoms, but confidence intervals produced were large given the small number of respondents reporting recent victimization. The significant findings were as follows: Recent non-violent victimization relative to no recent experience (OR 5.529, 95% CI 2.073–14.744), recent violent victimization with a firearm relative to no victimization ever (OR 6.162, 95% CI 1.615–23.498), and recent violent victimization without a firearm (OR 4.272, 95% CI 1.139–16.019) were all associated with higher odds of reporting feeling sad, blue or depressed. Recent violent victimization with a firearm also increased the likelihood that a person reported feeling anxious, tense or worried (OR 5.236, 95% CI 1.664, 16.475) relative to never experiencing a victimization, but neither recent non-violent victimization or violence without a firearm were associated with the odds of anxiety

Turning to secondary exposures, respondents who knew a friend or family member that had attempted or completed a suicide with a firearm were 2–3 times more likely than people not exposed to report moderately high levels of anxiety, depressive symptoms, and sleep difficulty. As an example, people who knew someone associated with a firearm suicide were more than 3 times more likely to report feelings of depressive symptoms (OR 3.089, 95% CI 1.329, 7.181), and the odds of reporting anxious feelings were 160% higher (OR 2.596, 95% CI 1.222, 5.516).

Regarding the impact of cumulative gun violence exposure, an increase in the number of firearm violence exposure types was associated with increases in the odds of self-reported poor health across the board in the range of 18 to 28% for each additional type of exposure. In supplementary analyses, exposure to friends that have attempted or completed suicides were also associated with a higher likelihood or more severe outcomes, measured as anxiety, depressive symptoms, or difficulty sleeping for at least 14 days or more in the past month.

We next employ an ordered logistic regression model to estimate the odds of experiencing a combination of all three mental health and sleep outcomes versus the combined other categories (Table 3), estimating the odds of experiencing moderate levels (7 days or more within the past month) of depressive symptoms, anxiety and sleep combined (Model 1), and the odds of experiencing severe levels (14 days or more within the past month) of the three outcomes combined.

**Table 3:** Ordered logistic regression estimates of experiencing poor combined depressive symptons, anxiety and sleep outcomes (*n* = 513)

Exposures	Moderate (7 days or more in past month)	Severe (14 days or more in past month)
	OR (SE) [95% CI]	OR (SE) [95% CI]
*Personal victim*		
Ever a victim of firearm violence	**2.043 (.518) [1.24–3.36]**	1.461 (.541) [.707–3.020]
Any attack w/out firearm	**1.489 (.249) [1.072–2.067]**	**1.626 (.409) [.992–1.664]**
Non-violent crime	**1.703 (.267) [1.251–2.317]**	1.361 (.309) [.872–2.125)
*Friends or family*		
Violent victim firearm	.955 (.208) [.623–1.463]	**1.972 (.612) [1.063–3.658]**
Firearm suicide	**3.384 (.897) [2.012—5.689]**	**2.984 (1.091) [1.457–6.108]**
Died by suicide (Any means)	1.021 (.245 [.638–1.633]	**1.961 (.611) [1.065–3.612]**
*Community*		
Heard about shooting	.992 (.241) [.616–1.596]	1.378 (.502) [.674–2.816]
Heard about suicide	1.475 (.325) [.956–2.273]	1.286 (.406) [.692–2.389]
*Cumulative*		
Total firearm exposures	**1.214 (.091) [1.048–1.406]**	**1.384 (.153) [1.115–1.720]**

All models control for respondent age, gender, race, ethnicity, veteran status, education, financial need, length of residence in the county, firearm ownership, concealed carry license, growing up with firearms in the home, membership in gun clubs, and firearm safety training

For respondents reporting ever being a victim of firearm violence, the odds of reporting moderate levels of all three outcomes versus zero, one or two are two times greater (OR 2.043, 95% CI 1.240–3.360). While violent victimization without a firearm was associated with severe levels of mental and sleep outcomes, firearm victimization was not.

Turning to indirect exposure to firearm violence, for exposure to firearm suicide among people close to them, the odds of reporting moderate levels of all three outcomes versus zero, one or two are 3.4 times greater (OR 3.384, 95% CI 2.012–5.689), holding all other variables constant. The odds of reporting severe levels of all three outcomes are 3 times greater (OR 2.984, 95% CI 1.457–6.108). For each additional firearm exposure, the odds of experiencing moderate levels of mental health and sleep difficulties are 1.2 times greater, and the odds of experiencing severe levels of mental health and sleep difficulties are 1.4 times greater (OR 1.384, 95% CI 1.115–1.720), holding all other variables constant (Table 4).

**Table 4:** Logistic regression estimates modeling drinking and drug use outcomes as a function of firearm violence exposure (*n* = 513)

Exposures	Model 1	Model 2
	Binge drinking weekly	Drugs concern
*Personal victim*		
Any incident w/a firearm	2.181 (0.886) [0.983–4.837]	1.393 (0.615) [0.586–3.309]
Any attack w/out firearm	**2.335 (0.994) [1.014–5.378]**	2.207 (0.938) [0.958–5.080]
Non-violent crime	**2.309 (0.944) [1.036–5.146]**	**3.220 (1.436) [1.344 – 7.715]**
*Friends or family*		
Violent victim	1.859 (0.725) [0.865–3.994]	1.405 (0.555) [0.648–3.046]
Firearm suicide	0.769 (0.395) [0.301–2.107]	**4.505 (2.094) [1.811—11.205]**
Died by suicide (Any means)	1.122 (0.483) [0.483–2.608]	0.885 (0.433) [0.339—2.309]
*Community*		
Heard about shooting	1.661 (0.706) [0.722–3.820]	1.436 (0.628) [0.609–3.385]
Heard about suicide	**3.909 (1.705) [1.662–9.191]**	0.547 (0.217) [0.251–1.193]
*Cumulative*		
Total firearm exposures	**1.343 (0.180) [1.033–1.747]**	1.117 (0.156) [0.849—1.470]

*All models control for respondent age, gender, race, ethnicity, veteran status, education, financial need, length of residence in the county, firearm ownership, concealed carry license, growing up with firearms in the home, membership in gun clubs, and firearm safety training

^+^Each of the personal victimizations were broken down into recent victimizations happening within the previous year, and earlier experiences. Recent victimization (relative to no recent experience, and relative to no experience ever) was associated with higher odds of reporting binge drinking and worrisome drug use, but confidence intervals produced were large given the small number of respondents reporting recent victimization. The significant findings were as follows: Recent non-violent victimization relative to no recent experience was associated with higher odds of reporting concerns about drug use (OR 3.123, 95% CI 1.258–7.751]. Recent victimization with a firearm (relative to no recent victimization) was associated with higher odds of reporting concerns with drug use (OR 4.435, 95% CI 1.259–15.628]

With respect to concerning drug use and binge drinking behavior, respondents that reported non-firearm and non-violent victimization were more likely than people who haven’t experienced a non-violent victimization to report more regular binge drinking episodes (OR 2.309, 95% CI 1.036–5.146) and report that someone has raised concern about their drug use (OR 3.220, 95% CI 1.344–7.715). Having a friend or family member associated with firearm suicide was also associated with a more than four-fold increase in the likelihood of concerning drug use. Although secondary suicide exposure was not associated with binge drinking in this sample, hearing about someone taking their own life with a firearm in the community at large was associated with an almost four-fold increase in the odds of frequent binge drinking.

## Discussion and conclusion

This study builds on a growing body of research that characterizes mental and physical effects of firearm exposure on individuals (Semenza et al., 2023a; Semenza et al., 2024a, 2024b; Smith et al., 2020; Song et al., 2022, 2023; Turner et al., 2019; Vasan et al., 2021) and within communities (Semenza & Stansfield 2021a; b; Semenza et al., 2023b). Specifically, it contributes novel evidence about firearm exposure among rural populations and illustrates how exposure in rural communities is associated with adverse behavioral and mental health effects. The study site offers a robust gun culture within which to study these questions, with gun ownership higher than estimates for gun ownership in rural America more generally based on other national surveys (e.g. Pew Research Center, 2017). As an example, 53% of respondents report personally owning at least one firearm in the sample, while national estimates for rural citizens are closer to 46% (Parker et al., 2017). Key takeaways in the community highlight the effects of suicidality among friends and family on respondent mental health and sleep outcomes, as well as the effects of cumulative firearm exposure on moderate and severe adverse health experiences.

Given the body of evidence that highlights the prevalence of firearm suicide in rural places (Lundstrom et al., 2023; Reeping et al., 2023), our results noting additional harmful effects of exposure to firearm suicide are important. Respondents who knew of someone who attempted or completed suicide with a firearm were 2–3 times more likely to experience anxiety, depressive symptoms and poor sleep. Interestingly, these associations were not significant for all completed suicides (nor for personal victimization without firearm or friend/family firearm victimization) and may reflect challenges in viewing mental health effects in isolation. Yet, when examining combined health outcomes, results show strong associations between knowing someone who attempted or completed firearm suicide and both moderate and severe health implications, as well as severe health implications for respondents who knew someone who died by any means of suicide. Moderate and severe effects might be explained by the rarity of mental health challenges occurring in isolation. Many individuals, especially those who have experienced trauma, experience co-occurring mental health challenges (Gatz et al., 2007; Mauritz et al., 2013; Stinson et al., 2016; Zarse et al., 2019).

Results around coping mechanisms associated with firearm suicide exposure highlighted that respondents knowing someone with a suicide firearm attempt or completion were 4.5 times more likely to have a drug use concern. This outcome could be explained by drug use to cope with complex interpersonal relationships and distress among individuals caring for a friend or family member experiencing suicidal behaviors (Marshall et al., 2023). Knowledge of firearm suicide within the community was associated with greater odds of binge drinking weekly, which could speak to social connections at alcohol serving establishments as well as maladaptive coping strategies involving alcohol use (Haden & Scarpa, 2008).

Additionally, our results showed the existence of an overall dose–response relationship, where exposure to additional experiences of firearm violence was associated with negative implications for mental and behavioral health. Cumulative firearm exposures were associated with greater odds of experiencing anxiety, depressive symptoms, and poor sleep, at both moderate and severe levels. Cumulative firearm exposures were also associated with greater odds of weekly binge drinking.

Some of the nonsignificant effects across results in the community firearm violence exposure and violent firearm exposure among friends or family might be explained by lack of direct knowledge or experience of the firearm incident(s). The stronger effects shown for firearm suicide exposure may speak to the frequency or prevalence of suicide within the county of study, or a weightier emotional limerence associated with knowledge or experience of a friend, family member, or community member attempting or completing suicide.

## Limitations

This study is not without limitations. Perhaps the biggest drawback was our use of a non-probability sampling method. We acknowledge that people who spend limited time online or exposed to social media and advertising will have been less likely to complete the survey. Post stratification weights can help undo some distortions associated with non-probability samples, although weighting non-probability samples will only produce unbiased estimates for all traits if the response propensities are equal across groups (Salganik, 2019). There are also natural challenges with survey quality control and ensuring that respondents were residents of the county, beyond the use of IP address locations and reported geographical identifiers. Given our conservative approach, it’s more likely that a county resident was wrongly excluded from analysis, rather than a non-resident being included.

There are several other important limitations. First, because data are cross-sectional, causality cannot be established between variables. Another limitation is related to temporal ambiguity and differences in recency of variables. The health outcome variables refer to the past 30 days, while the firearm and violence exposure variables address lifetime (if ever) exposure. As noted in the footnotes of Table 2, personal victimization variables were available in recent (past 12 months) and earlier experiences, yet small response rates precluded them from the main analyses. Future analyses can more accurately measure recent and frequency of firearm exposure and align these measures with the recent and frequency of outcome variables. The self-report nature of the data is subject to respondent recall error and other responses biases. This study focused on one county in rural Pennsylvania, and future research can expand inquiry into multi-county, statewide or national rural samples.

## Implications for policy and practice

While interpersonal firearm violence occurs in rural places to a greater extent than mainstream narratives might suggest, rural firearm violence is mainly driven by firearm suicides (Pierce et al., 2024; Reeping et al., 2023). Numerous research outlets have chronicled a web of related factors associated with firearm suicide in rural places—lost social connections, heightened opioid use, burgeoned chronic illnesses, and plateaued or diminished economic opportunity (Kalesan, 2020; Lundstrom et al., 2023; Shiner et al., 2022). One avenue to counter the effects of cumulative firearm exposure and firearm suicide exposure is through reinvigorating community spaces and strengthening social bonds. More robustly resourced programs that support secure firearm storage and social connections in rural communities are needed (Pollock et al., 2020). Avenues for social support not only buffer against deteriorating mental health but provide pro-social activities and support (informal or formal) for individuals who have been exposed to firearm violence and suicide (Haden & Scarpa, 2008). Support and social groups can be connected through local churches, gun shops/ranges, and businesses (University of Colorado Boulder, 2024). Shared social spaces enable residents to connect and heal and can engender positive coping skills that cushion potentially destructive alcohol or drug use (Barnard et al., 2022; Pollock et al., 2020).

## Conclusion

This cross-sectional study outlines associations between interpersonal and self-directed firearm violence exposure and self-rated behavioral and mental health and sleep disturbance outcomes among residents in a rural Pennsylvania county. Knowing someone associated with firearm suicide is related to harmful health outcomes. Cumulative firearm exposure is also associated with deleterious health outcomes. The results support prior research outlining the damaging effects of firearm exposure on individuals and contributes new information highlighting the particularly harmful effect of firearm suicide exposure on health within a rural population.

## Data Availability

Data are owned by the local county government and not available for public use.

## Appendix 1: Survey Questions Used for Data Analysis

**Table:**

Survey questions used in the study		Response categories
*Exposure to gun violence*		
Have you (ever/past year) been the victim of…?	Attack with a weapon	Yes, No
	Attack without a weapon	Yes, No
	A nonviolent crime	Yes, No
Has someone close to you or someone in your family ever….?	Experienced a violent victimization by a stranger with a firearm	Yes, No
	Died by suicide with a firearm	Yes, No
	Attempted suicide with a firearm	Yes, No
	Attempted suicide without a firearm	Yes, No
	Completed a suicide without a firearm	Yes, No
Have you ever witnessed or heard about someone being shot in your community?		Yes, No
Have you ever witnessed or heard about someone dying by suicide by firearm in your community?		Yes, No
*Firearms history*		
Did you grow up with firearms in the home?		Yes, No
Do you currently participate in any social activities related to firearms such as sports shooting clubs or collector groups?		Yes, No
Have you received formal training on firearm safety?		Yes, including suicide prevention; Yes without suicide prevention; No
Do you currently own any firearms?		Yes (Enter Estimated Number), No
Are you currently licensed to carry a concealed firearm publicly in PA?		Yes, No
*Health and anxiety*		
During the past 30 days, for about how many days have you felt sad, blue, or depressed?		0 days; 1–3 days; 3–7 days; 7–14 days; 14 or more days
During the past 30 days, for about how many days have you felt WORRIED, TENSE, or ANXIOUS?		0 days; 1–3 days; 3–7 days; 7–14 days; 14 or more days
During the past 30 days, for about how many days have you felt you did NOT get enough rest or sleep?		0 days; 1–3 days; 3–7 days; 7–14 days; 14 or more days
How many times during the past 30 days did you have [5 or more for men, 4 or more for women] drinks on an occasion?		Never; Once or twice; Once a week; more than weekly; Daily or almost daily
During the past 30 day, has someone told you that they were concerned about your use of drugs?		Yes, No
*Demographics*		
How much do you agree that you have enough money to meet your needs?		Strongly Disagree (1)—Strongly Agree (5)
What is your gender?		Male, Female, Other
What is your age?		Enter Years
What is your race and/or ethnicity?		White or Caucasian; Black of African American; Hispanic or Latino; Asian; American Indian or Alaska Native; Native Hawaiian or Other Pacific Islander; Two or more races; Other (please specify)
Do you have any children or youth under the age of 18 in your household?		Yes, No
What level of education have you completed?		Less than high school; high school; some college; Bachelor's or Associate; Graduate degree or higher
What is your marital status?		Never married; married; separated; divorced; widowed
Have you ever served in the military or armed forces?		Yes, No
How long have you lived in {} County?		Years and Months
